# Infant formula containing large, milk phospholipid-coated lipid droplets and dairy lipids affects cognitive performance at school age

**DOI:** 10.3389/fnut.2023.1215199

**Published:** 2023-09-05

**Authors:** Lidewij Schipper, Nana Bartke, Maya Marintcheva-Petrova, Stefanie Schoen, Yvan Vandenplas, Anita C. S. Hokken-Koelega

**Affiliations:** ^1^Danone Nutricia Research, Utrecht, Netherlands; ^2^Universitair Ziekenhuis (UZ) Brussel, Brussel, Belgium; ^3^Erasmus Medisch Centrum -Sophia Kinderziekenhuis, Rotterdam, Netherlands

**Keywords:** infant, nutrition, lipids, erythrocyte, cognition, PUFA, NIH toolbox

## Abstract

**Background:**

Breastfeeding has been positively associated with infant and child neurocognitive development and function. Contributing to this effect may be differences between human milk and infant formula in the milk lipid composition and milk fat globule structure.

**Objective:**

To evaluate the effects of an infant formula mimicking human milk lipid composition and milk fat globule structure on childhood cognitive performance.

**Methods:**

In a randomized, controlled trial, healthy term infants received until 4 months of age either a Standard infant formula (*n* = 108) or a Concept infant formula (*n* = 115) with large, milk phospholipid coated lipid droplets and containing dairy lipids. A breastfed reference group (*n* = 88) was included. Erythrocyte fatty acid composition was determined at 3 months of age. Neurocognitive function was assessed as exploratory follow-up outcome at 3, 4, and 5 years of age using the Flanker test, Dimensional Change Card Sort (DCCS) test and Picture Sequence Memory test from the National Institutes of Health Toolbox Cognition Battery. Mann–Whitney U test and Fisher exact test were used to compare groups.

**Results:**

Erythrocyte omega-6 to -3 long-chain polyunsaturated fatty acid ratio appeared to be lower in the Concept compared to the Standard group (*P* = 0.025). At age 5, only the Concept group was comparable to the Breastfed group in the highest reached levels on the Flanker test, and the DCCS computed score was higher in the Concept compared to the Standard group (*P* = 0.021).

**Conclusion:**

These outcomes suggest that exposure to an infant formula mimicking human milk lipid composition and milk fat globule structure positively affects child neurocognitive development. Underlying mechanisms may include a different omega-3 fatty acid status during the first months of life.

**Clinical trial registration:**

https://onderzoekmetmensen.nl/en/trial/28614, identifier NTR3683 and NTR5538.

## Introduction

Breastfeeding has been positively associated with many infant and child health outcomes including neurocognitive development and function (1, 2). Amongst the factors likely contributing to these effects are differences between human milk (HM) and infant formula (IF) in dietary lipid quality. Omega (n)-3 and n-6 long-chain polyunsaturated fatty acids (LCPUFAs) in HM including docosahexaenoic acid (DHA, 22:6n-3) accumulate rapidly in neuronal membranes during early life, depending on feeding mode (3, 4). Moreover, child neurocognitive outcomes have been associated with dietary (LC)PUFA supply in the first year of life (5, 6). Lipids in IF differ from those in HM in composition and structure (7). HM comprises large lipid globules (mode diameter of ∼4 μm) that are encapsulated by the milk fat globule membrane (MFGM), a 3-layered membrane composed of phospholipids (PL), sphingolipids, glycolipids, proteins and other components. The human milk lipid globule is generated in the mammary gland epithelial cells. Triglycerides are synthesized in the endoplasmatic reticulum and are thereafter released into the cytosol in small lipid droplets, surrounded by a single layer membrane composed of polar lipids and membrane proteins. These small lipid droplets fuse to form large lipid globules, that are then enveloped by the double layered plasma cell membrane upon secretion from the mammary gland cell, forming together the MFGM (8). In contrast, infant formulas are produced in the factory, and are often based on bovine milk based protein ingredients and mostly vegetable oils providing the lipid fraction. Vegetable oils do no form lipid droplets similar to those seen in mammalian milk. While lipid globules in raw bovine milk are also large and encapsulated by a similar MFGM, this natural membrane structure is disrupted during common manufacturing processes such as homogenization and emulsification, and the membrane components are lost due to fractionation and removal (9). As a result, the lipid droplets in IF are usually small (mode diameter of <0.5 μm) with no phospholipid membrane and only milk proteins adhering to their surface (10). Recent developments in IF include the addition of dairy lipids and MFGM (fragments) enriched ingredients that bring IF lipid composition closer to that of HM (11–13). Importantly, additional similarities to HM are generated by adaptations in the IF manufacturing process, resulting in IF with lipid droplets large in size (mode diameter of ∼3–5 μm) and coated by PL, mimicking more closely the structural properties of HM lipid globules (10). These structural properties are of functional relevance as the lipid droplet size and the presence of the phospholipid membrane have been shown to affect digestion and absorption kinetics of lipid droplets after ingestion and their postprandial appearance in the bloodstream (14–18). The subsequent bioavailability of lipids and postprandial hormones to the developing brain is hypothesized to influence infant neurocognitive development (7).

In preclinical experiments using mouse models, early life exposure to Concept compared to Standard IF resulted in long term improvement in metabolic health and neurocognitive outcomes (19–21). In a randomized, controlled trial (Dutch Trial Register; NTR3683), we demonstrated that a Concept IF with dairy lipids and comprising large lipid droplets coated by (bovine MFGM derived) PL supported adequate growth and was safe and well-tolerated in healthy term infants (22). A long-term follow-up of the same study (Dutch Trial Register; NTR5538) followed the participants until 5 years of age, with growth and body composition development as primary outcome parameters. Here, we report on the effects of the Concept IF on infant erythrocyte fatty acid composition and cognitive outcomes during childhood that were exploratory parameters in these trials. It was hypothesized that infants exposed to Concept IF would show improved neurocognitive outcomes in early childhood compared to infants that were exposed to standard IF.

## Materials and methods

### Participants and study design

The Mercurius study is a randomized, double-blind, controlled, prospective, multi-country trial, of which the study design and in- and exclusion criteria have been previously reported in detail (22). In short, healthy, term infants were randomized until (≤ ) 35 days of age to receive either Standard IF (Standard group, *n* = 108) or Concept IF (Concept group, *n* = 115) until 17 weeks of age. Infants whose mothers intended to exclusively breastfeed until at least 13 weeks of age were included as a non-randomized reference group (Breastfed group, *n* = 88). The formulas (Table 1) were similar in energy content, total lipid content and n-3 and -6 PUFA composition, but in the Concept IF the vegetable oil fraction was partially replaced by dairy lipids (48%), and milk PL derived from bovine MFGM were added. Detailed information about fatty acid composition in the formula is available in Supplementary Table 1. Importantly, lipid droplets in the Concept IF were large (mode diameter of ∼3–5 μm) and were coated with (bovine MFGM derived) PL due to an altered production process, while lipid droplets in the Standard IF were small (mode diameter of ∼0.5 μm) with only milk proteins adhering to the surface. At 13 weeks of age, a voluntary blood sample was drawn via heel prick from infants whose caregivers gave consent for this procedure. If the infant had completed the intervention period until 17 weeks of age, its caregivers were contacted for participation in the Mercurius follow-up study. Cognitive function of the children was assessed at 3, 4, and 5 years of age. Investigators and parents were unblinded after database lock of the follow-up study.

**TABLE 1:** Composition of the intervention products.

			Standard IF	Concept IF
**Structural properties of lipid droplets**				
	Mode diameter	μ m	∼0.5	∼3–5
	Surface coated by PL		No	Yes
**Composition (per 100 ml)**				
Energy		kcal	66	66
Protein		g	1.3	1.3
Carbohydrates		g	7.3	7.3
Fat		g	3.4	3.4
	Vegetable oil	g	3.3	1.7
	Dairy lipids	g	0.1	1.6
	Milk phospholipids	Mg	–	55
	Soy phospholipids	mg	4.5	–
**Fatty acids (per 100 ml)**				
Saturated (SFA)		g	1.5	1.4
Monounsaturated (MUFA)		g	1.3	1.2
Polyunsaturated (PUFA)		g	0.6	0.6
	C18:2n-6 Linoleic (LA)	mg	447	447
	C18:3n-3 Alpha-linolenic (ALA)	mg	82	83
	C20:4n-6 Arachidonic acid (ARA)	mg	11	12
	C20:5n-3 Eicosapentaenoic (EPA)	mg	1.4	1.8
	C22:6n-3 Docosahexaenoic acid (DHA)	mg	6.4	6.6

### Erythrocyte fatty acids

Approximately 500 μL blood was drawn from non-fasted infants via heel prick at 13 weeks of age. The blood was collected in heparin tubes (Microtainer tubes, Becton Dickinson) and centrifuged for 3 min at 2,000 g. After removal of plasma the erythrocyte fraction was stored at −80°C. Erythrocyte fatty acids (FA) were analyzed at Nutricia Research (Utrecht, The Netherlands) by means of gas chromatography with flame ionization detection (GC-FID). Erythrocyte lipids were extracted according to a modified procedure of Bligh and Dyer (23). In a glass tube, 2 ml of 1% EDTA solution was added then 150 μl of erythrocyte fraction was added and then vortexed. After that 2 ml of methanol was added and solution was vortexed again. After adding dichloromethane the solution was vortexed for 5 min and then 10 min centrifuged at 3,000 rpm. The dichloromethane layer, containing the lipids, was collected in a new glass tube and then evaporated to dryness using a SpeedVac^®^. The dried lipids were converted to fatty acid methyl esters (FAME) by adding 2 ml methanol and 40 μl concentrated sulfuric acid and heated at 100°C for 60 min (24). After cooling down, 2 ml hexane and 0.5 ml 2.5 mol/l sodium hydroxide solution was added to the glass tubes and then vortexed for 2 min. The hexane layer, containing the FAME’s, was collected in a new glass tube and then evaporated to dryness using a SpeedVac^®^. Dried samples were subsequently dissolved in 150 μl iso-octane and analyzed by GC-FID with a CP-SIL88 for FAME column (50 m × 0.25 mm id. 0.22 μm film thickness). The FAME’s were identified based on retention time using an external reference standard GLC-461 and GLC-68D and loose standard of C22:5n-6 (all Nu-Chek Prep.). Peak area was used to measure of relative percent of individual fatty acids. Erythrocyte fatty acid concentration was expressed as percentage of total fatty acids.

### Cognitive function

At 3, 4, and 5 years of age, cognitive function of children was assessed using the National Institutes of Health Toolbox Early Childhood Cognition Battery (NHITB-CB) iPad app (English, version 1.8) (25). Three selected tests from the toolbox were administered focusing on measures of inhibitory control and selective attention (Flanker Inhibitory Control and Attention test, FICA), cognitive flexibility (Dimensional Change Card Sort test, DCCS) and episodic memory function (Picture Sequence Memory test, PSM) (26, 27). The tests were administered by a trained child psychologist. Each toolbox test included trials at practice level and trials at test level(s). Children progressed to the (next) test level only after successfully completing previous practice (and test) levels. For each subject, the highest reached level during the toolbox test (e.g., “practice”) was derived, and only for subjects passing practice level, the computed score per toolbox test (FICA, DCCS, PSM) was analyzed. Data of 2 subjects in the Standard and of 2 subjects in the Concept group were excluded from all analyses, as their performance could have been potentially affected by their medical condition (Medical history/Adverse event, identified during a data review meeting before data analyses). One subject in the Standard group received assistance from his/her caregiver during the tests and was also excluded from analyses.

### Statistical analyses

Group differences in erythrocyte fatty acid composition were investigated using the Mann-Whitney U test. Differences in the medians of groups and 95% confidence intervals (CI) were calculated for each pair of groups using bootstrapping re-sampling method (with 50,000 samples taken with replacement) and applying bias-correction and acceleration (BCa method) using Jackknife method. Highest reached levels of the NIH tests were compared within each group between consecutive years using the Bhapkar marginal homogeneity test, and for each year between the (pair of) groups using Fisher exact test. Computed scores were compared within each group between consecutive years using the Wilcoxon signed-rank test, and for each year between the (pair of) groups using Mann–Whitney U test. All data were analyzed using the available (non-missing) data from the subjects in the intention to treat (ITT) population.

## Results

### Study population

**FIGURE 1:**
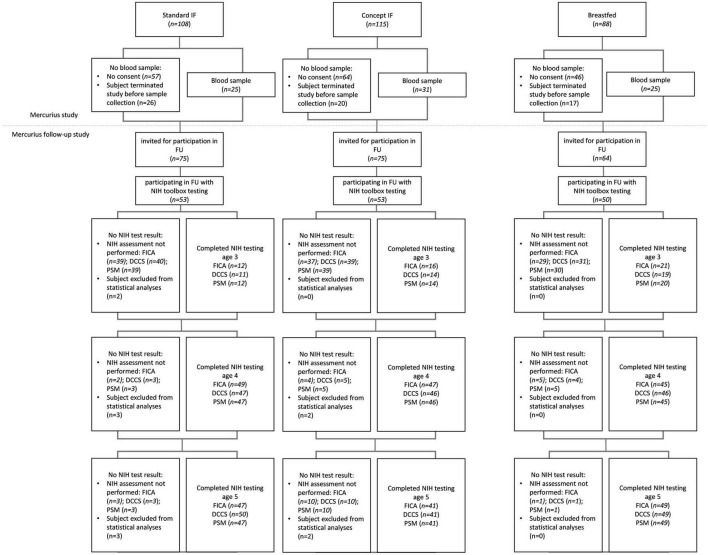
Consort diagram.

Enrolment in the Mercurius study took place from October 2012 to December 2013 and enrolment in the Mercurius follow-up study was between January 2016 and August 2018. Erythrocyte fatty acid composition data was available for 81 subjects (Standard *n* = 25; Concept *n* = 31; Breastfed *n* = 25). A total of 156 subjects participated in NIH toolbox testing between 3 and 5 years of age, of which 151 were included in the analyses presented here (Standard, *n* = 50; Test, *n* = 51; Breastfed *n* = 50). Details are listed in Figure 1. Baseline characteristics of infants with erythrocyte fatty acid composition data and of children participating in the NIH toolbox assessment of the follow-up study are presented in Supplementary Table 2.

### Erythrocyte fatty acid composition

Table 2 shows the relative concentration of (selected) fatty acids in erythrocyte membranes per study group. Erythrocyte total saturated fatty acids (SFA) in the Concept group was comparable to that of the Standard group and closer to that of Breastfed infants. The two IF groups were close in total erythrocyte mono-unsaturated fatty acids (MUFA), but erythrocyte PUFA levels were higher in the Concept compared to the Standard group, albeit not statistically significantly different. Total n-6 and n-3 LCPUFA levels were comparable between the IF groups. The Concept group showed a lower erythrocyte n-6 to n-3 LCPUFA ratio when compared to the Standard group. In contrast, the Standard group presented a higher erythrocyte n-6 to n-3 LCPUFA ratio when compared to the Breastfed group. Amongst the individual n-6 LCPUFA species, dihomo-gamma linolenic acid (DGLA, 20:3n-6) was lower in the Concept group compared to Standard IF and to Breastfed infants. Adrenic acid (22:4n-6) appeared to be higher in the Standard group compared to Breastfed infants, and a similar effect was observed for Concept compared to Breastfed infants. N-6 docosapentaenoic acid (n6DPA, 22:5n-6) was higher in the Standard compared to the Breastfed group. Erythrocyte alpha-linolenic acid (ALA, 18:3n-3) levels were higher in both formula fed groups compared to the Breastfed reference. Eicosapentaenoic acid (EPA, 20:5n-3) was higher in the Concept group than in the Standard group and closer to Breastfed group. Higher levels of n-3 docosapentaenoic acid (n3DPA, 22:5n-3) were present in Concept compared to Standard group, although the difference was not statistically significant.

**TABLE 2:** Erythrocyte fatty acid concentrations as a percent of total fatty acids (wt/wt) at 13 weeks of age.

Fatty acids	Median (Q1–Q3)			Median difference (95% CI)^1^; *p*-value^2^		
	Standard (*N* = 25)	Concept (*N* = 31)	Breastfed (*N* = 25)	Standard vs. concept	Concept vs. breastfed	Standard vs. breastfed
Saturated (SFA)	37.47 (36.61–38.23)	37.93 (36.66–40.35)	38.97 (37.35–40.27)	0.46 (−0.82; 1.39); 0.391	−1.04 (−2.51; 0.45); 0.315	−1.50 (−2.56; −0.12); 0.052
Monounsaturated (MUFA)	27.93 (26.55–29.56)	27.72 (25.33–29.63)	26.53 (24.63–30.13)	−0.21 (−2.09; 1.56); 0.410	1.19 (−1.52; 3.36); 0.692	1.40 (−1.60; 3.31); 0.252
Polyunsaturated (PUFA)	32.49 (31.63–33.36)	33.72 (30.70–34.54)	32.37 (30.11–33.90)	1.22 (0.76; 2.03); 0.054	1.35 (−0.03; 2.62); 0.166	0.12 (−1.36; 1.44); 0.801
18:2n-6 Linoleic (LA)	13.31 (12.86–14.38)	13.68 (12.55–15.78)	13.31 (12.01–15.27)	0.37 (−0.41; 1.76); 0.323	0.37 (−0.91; 2.06); 0.609	−0.00 (−1.43; 1.13); 0.877
18:3n-3 α-linolenic (ALA)	0.46 (0.35–0.60)	0.44 (0.34–0.65)	0.29 (0.20–0.40)	−0.019 (−0.182; 0.098); 0.479	0.151 (0.017; 0.272); **<0.001**	0.170 (0.077; 0.311); **<0.001**
20:2n-6 Eicosadienoic	0.49 (0.44–0.56)	0.47 (0.43–0.51)	0.48 (0.44–0.54)	−0.021 (−0.078; 0.044); 0.171	−0.017 (−0.060; 0.029); 0.277	0.004 (−0.076; 0.062); 0.816
20:3n-6 Dihomo-gamma linolenic (DGLA)	1.04 (0.87–1.15)	0.91 (0.74–1.01)	1.11 (0.91–1.31)	−0.130 (−0.298; 0.023); **0.05**	−0.204 (−0.384; −0.018); **0.004**	−0.074 (−0.255; 0.145); 0.174
20:4n-6 Arachidonic (ARA)	9.56 (9.18–10.65)	9.28 (7.74–11.19)	8.95 (7.19–10.50)	−0.28 (−1.59; 1.05); 0.531	0.33 (−1.29; 2.02); 0.448	0.61 (−0.76; 1.96); 0.181
22:4n-6 Adrenic	1.94 (1.55–2.14)	1.65 (1.02–2.02)	1.24 (0.96–1.63)	−0.290 (−0.803; 0.112); 0.138	0.407 (−0.001; 0.933); 0.052	0.697 (0.353; 1.133); **<0.001**
20:5n-3 Eicosapentaenoic (EPA)	0.28 (0.23–0.32)	0.40 (0.32–0.46)	0.41 (0.25–0.51)	0.121 (0.074; 0.179); **<0.001**	−0.009 (−0.092; 0.144); 0.818	−0.131 (−0.191; 0.029); **0.017**
22:5n-3 Docosapentaenoic (n3DPA)	0.86 (0.77–0.99)	1.01 (0.70–1.33)	0.94 (0.65–1.12)	0.149 (−0.036; 0.348); 0.072	0.072 (−0.167; 0.290); 0.242	−0.077 (−0.258; 0.076); 0.426
22:5n-6 Docosapentaenoic (n6DPA)	0.36 (0.30–0.38)	0.31 (0.18–0.37)	0.25 (0.20–0.34)	−0.050 (−0.157; 0.003); 0.099	0.061 (−0.029; 0.116); 0.575	0.111 (0.054; 0.152); **0.021**
22:6n-3 Docosahexaenoic (DHA)	3.18 (2.81–3.50)	3.31 (2.16–3.57)	3.67 (2.82–4.32)	0.13 (−0.60; 0.57); 0.729	−0.37 (−1.38: 0.41); 0.121	−0.49 (−1.24; 0.27); 0.151
Total n-6 long chain PUFA	13.47 (12.54–14.63)	12.74 (10.26–15.26)	12.17 (9.69–14.16)	−0.72 (−3.22; 1.07); 0.315	0.57 (−1.69; 2.71); 0.510	1.30 (−0.47; 3.78); 0.091
Total n-3 long chain PUFA	4.35 (4.12–4.85)	4.75 (3.21–5.49)	4.89 (3.66–5.90)	0.40 (−0.62; 0.96); 0.356	−0.14 (−1.36; 1.10); 0.255	−0.54 (−1.42; 0.56); 0.081
n6/n3 long chain PUFA	3.14 (2.93–3.41)	2.93 (2.70–3.17)	2.72 (2.40–3.02)	−0.214 (−0.464; 0.016); **0.025**	0.206 (−0.071; 0.488); 0.056	0.419 (0.200; 0.805); **<0.001**

^1^Estimated differences between the medians and 95% CIs were calculated using bootstrapping re-sampling method (with 50,000 samples taken with replacement) and applying bias-correction and correcting for acceleration (BCa method) using Jackknife method.

^2^*P*-value obtained by Mann–Whitney U test. *P*-values below 0.05 are in bold.

### Cognitive function

**FIGURE 2:**
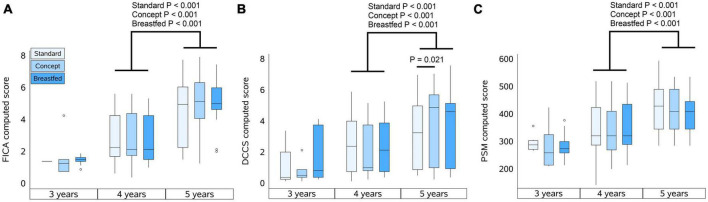
Computed scores of NIH tests at 3, 4, and 5 years of age. **(A)** Flanker Inhibitory Control and Attention test (FICA), **(B)** Dimensional Change Card Sort test (DCCS), and **(C)** Picture Sequence Memory test (PSM). Box plots per group represent the median, upper and lower quartiles and the whiskers extend to the most extreme value within 1.5 times the interquartile range (IQR) with scores that are at a distance of more than 1.5*IQR from the box indicated by markers. Computed scores were compared within each group between 4 and 5 years of age using the Wilcoxon signed-rank test, and for each year between groups using Mann–Whitney U test.

When the follow-up study started, the preparations required to perform the cognition tests were not fully finalized. As a result, the number of subjects that were subjected to cognitive testing at the 3 years timepoint was limited. The total number of subjects per group subjected to cognitive testing at each timepoint is shown in Table 3. The highest reached level for FICA, DCCS, and PSM are summarized in Table 3 for each study group at 3, 4, and 5 years of age, and the computed scores for each test are presented in Figures 2A–C, respectively.

**TABLE 3:** Highest reached levels in NIH tests at 3, 4, and 5 years of age.

	Highest reached levels (%)			*P* ^1^		
	**Standard**	**Concept**	**Breastfed**	**S vs. C**	**C vs. B**	**S vs. B**
**Age 3**						
FICA	(*n* = 12)	(*n* = 16)	(*n* = 21)			
practice	83.3	56.3	52.4			
Fishes	16.7	37.5	47.6			
Arrows	0.0	6.3	0.0	0.308	0.498	0.133
DCCS	(*n* = 11)	(*n* = 14)	(*n* = 19)			
Practice	27.3	28.6	26.3			
Pre-switch (color)	36.4	35.7	21.1			
Post-switch (shape)	18.2	28.6	26.3			
Mixed	18.2	7.1	26.3	0.941	0.605	0.845
PSM	(*n* = 12)	(*n* = 14)	(*n* = 20)			
practice	33.3	42.9	30.0			
Test	66.7	57.1	70.0	0.701	0.487	>0.999
**Age 4**						
FICA	(*n* = 48)	(*n* = 47)	(*n* = 45)			
Practice	16.7	17.0	15.6			
Fishes	45.8	48.9	44.4			
Arrows	37.5	34.0	40.0	0.937	0.839	0.968
DCCS	(*n* = 47)	(*n* = 46)	(*n* = 46)			
Practice	2.1	4.3	0.0			
Pre-switch (color)	8.5	6.5	4.3			
Post-switch (shape)	31.9	45.7	45.7			
Mixed	57.4	43.5	50.0	0.498	0.633	0.386
PSM	(*n* = 47)	(*n* = 46)	(*n* = 45)			
Practice	17.0	15.2	13.3			
Test	83.0	84.8	86.7	0.813	0.797	0.623
Change from 3 to 4		*P* ^2^				
FICA	**<0.001**	**<0.001**	**<0.001**			
DCCS	**<0.001**	**<0.001**	**0.003**			
PSM	**0.046**	**0.026**	0.078			
**Age 5**						
FICA	(*n* = 47)	(*n* = 41)	(*n* = 49)			
Practice	0	2.4	4.1			
Fishes	29.8	19.5	10.2			
Arrows	70.2	78	85.7	0.328	0.474	**0.019**
DCCS	(*n* = 47)	(*n* = 41)	(*n* = 49)			
Practice	0.0	0.0	2.0			
Pre-switch (color)	0.0	4.9	4.1			
Post-switch (shape)	42.6	24.4	30.6			
Mixed	57.4	70.7	63.3	0.065	0.892	0.249
PSM	(*n* = 47)	(*n* = 41)	(*n* = 49)			
Practice	2.1	0.0	4.1			
Test	97.9	100.0	95.9	>0.999	0.498	>0.999
Change from 4 to 5		*P* ^2^				
FICA	**<0.001**	**<0.001**	**<0.001**			
DCCS	0.196	**0.003**	0.232			
PSM	**0.004**	**0.007**	**0.049**			

^1^*P*-values obtained by Fisher exact test (between groups per year).

^2^*P*-values obtained by Bhapkar marginal homogeneity test (within each group between consecutive years). *P*-values below 0.05 are in bold. DCCS, Dimensional Change Card Sort test; FICA, Flanker Inhibitory Control and Attention test; PSM, Picture Sequence Memory test.

#### FICA

At 3 years of age the majority of the children who were subjected to FICA did not pass the practice level and also at 4 years of age not all children passed practice. At 5 years, however, most children did pass the practice level, and each group showed an improvement in highest reached levels and computed scores (*P* < 0.001 for all groups) with increasing age. The highest reached levels at 3 years of age in Standard group appeared to be different as compared to Concept and Breastfed groups, but this was not statistically significant. At 4 years of age the highest reached levels were similar between the groups. At 5 years of age, however, the highest level reached by children in the Breastfed group was higher than that of the Standard group, but not (much different as compared to) the Concept group. Computed scores at 5 years of age were comparable between the groups.

#### DCCS

In each group, a similar proportion of children who were subjected to DCCS did not pass the practice level at 3 and 4 years of age. All groups showed improvement in highest reached levels between 3 and 4 years of age, and from the IF groups only the Concept group also showed an improvement in highest reached levels between 4 and 5 years. All groups showed an increase in computed scores between 4 and 5 years of age (Standard, *P* = 0.05; Concept, *P* < 0.001; Breastfed *P* < 0.001). At 5 years of age, the highest levels reached in the Concept group were not different to those of Breastfed group and appeared to be higher than in the Standard group, albeit not statistically significantly different, but the computed score of the Concept group was significantly higher than that in the Standard group.

#### PSM

Not all children passed the practice level at 3 and 4 years of age. The highest reached levels at 4 years were higher compared to the previous year in all groups, albeit not statistically significantly for the Breastfed group. Between 4 and 5 years of age, all groups showed improvement in highest reached levels and in computed scores (Standard, *P* < 0.001; Concept, *P* < 0.001; Breastfed *P* = 0.005). The highest reached levels and the computed scores were, however, comparable between study groups at all ages.

## Discussion

Children who were breastfed during infancy typically show better neurocognitive function compared to formula fed children (1, 28, 29). Here we present, for the first time, data suggesting that exposure to IF that resembles HM in both lipid composition and structure (i.e., containing large, milk PL-coated lipid droplets with dairy fat) for 3–4 months during infancy may improve cognitive function during childhood, bringing performance closer to the levels observed after exclusive breastfeeding. These exploratory clinical results are in line with previous preclinical experiments using rodent models in which early life exposure to a similar Concept IF improved cognitive performance, with beneficial effects persisting into adulthood (20, 30).

At 5 years of age, children exposed to Concept IF appeared to show higher DCCS scores as compared to Standard IF, and children exposed to Concept IF, but not Standard IF, reached FICA levels comparable to the Breastfed group. The DCCS and FICA both measure executive function (26), which is considered an important predictor of later academic achievement (31, 32). Moreover, improved cognitive performance during childhood is generally believed to be associated with better mental and physical health, as well as financial and professional success, throughout life (33, 34). Interestingly, age-related changes in cognitive performance may not always be linear (35) and for formula fed infants, improvement during childhood has been shown to depend on IF dietary lipid quality. In one study, infants exposed to IF supplemented with DHA and arachidonic acid (ARA) showed age-related improvements in DCCS performance between 36 and 60 months of age, but infants that received a product without LCPUFA did not show any improvement (6). Likewise, in our study, children exposed to Concept IF, but not Standard IF, showed age-related improvements in DCCS performance between 4 and 5 years of age. The PSM test has previously been shown sensitive to detect age-related changes in episodic memory (27). While there were no differences between groups in PSM outcome in the current study, this does not negate the existence of differences in overall cognitive function between groups. Other studies have reported memory function specifically not being affected by early life feeding mode while other cognitive functions were (1, 36).

The underlying mechanisms by which the Concept IF may improve child cognitive function may include improved n-3 LCPUFA accumulation in the brain during the first year of life. At 13 weeks of age, we observed a lower n-6 to n-3 LCPUFA ratio in erythrocyte membranes of infants exposed to Concept IF compared to Standard IF. Erythrocyte n-6 and -3 LCPUFA composition is considered an indirect biomarker for LCPUFA composition in neuronal tissue as evident from positive correlations between erythrocyte and brain n-3 LCPUFA in tissue derived from infants after death (3) and data from animal models, e.g., (37, 38). In line with this, several studies have reported higher erythrocyte n-3 LCPUFAs during infancy to be predictive of better neural and visual function later in life (39, 40).

Infant n-3 FA status is well-known to be directly influenced by the supply of preformed n-3 and -6 LCPUFAs, as well as their precursors LA and ALA (41). While the Concept and Standard IFs in the current study were similar in n-3 and -6 fatty acid composition, part of the lipid fraction in Concept IF was derived from dairy lipids rather than vegetable oil, resulting in more efficient absorption of sn-2 palmitic acid (PA C16:0) in the Concept compared to Standard group (42–44). Moreover, short–and medium chain fatty acids including butyric acid (C4:0), caproic acid (C6:0) and caprylic acid (C8:0) were slightly higher in the Concept IF compared to Standard IF. It has been proposed that a higher supply of these fatty acids would spare relatively more ALA from rapid oxidation after absorption, thereby supporting endogenous conversion of ALA to n-3 LCPUFA and promoting incorporation of n-3 LCPUFA in tissue membranes (45, 46). In line with this, infants consuming an IF containing a mixture of dairy lipids and vegetable oil were shown to have higher erythrocyte n-3 LCPUFAs compared to infants who were fed an IF containing vegetable oil only (45). Using animal models, several studies confirmed higher n-3 PUFAs in brain tissue as a result of dairy lipid exposure (38, 46, 47), but others did not (48), and the effects on functional outcomes such as neuroplasticity and cognition appear to be mixed (47, 49, 50). Currently, the evidence is insufficient to confirm a role of sn-2 PA supplementation on infant neurodevelopmental outcomes (51).

In addition to dairy lipids, the concept IF contained milk PL sourced from bovine MFGM. Several clinical trials have demonstrated that MFGM or specific MFGM-derived lipid components as added ingredients to infant nutrition during the first year of life can be associated with improved cognitive, language and motor outcomes in infancy (52–55), with some (56, 57) but not all (58) studies showing effects to be sustained for a longer period. Despite additional differences in composition of the study products used [i.e., the MFGM supplemented IF was lower in protein (52), contained one or more other potentially bioactive ingredients such as lactoferrin (53), synbiotics and LCPUFAs (56), or had lowered LA/ALA ratio (55)], MFGM was suggested to be responsible for the neurocognitive benefits observed in these studies. Some of the lipid components present in MFGM such as sphingomyelin (SM) or gangliosides and other PL can also be found in (neuronal) cell membranes, and elevated plasma or serum levels of these have been reported following MFGM supplementation in the aforementioned trials (54, 55, 59). It may be plausible that higher levels of plasma SM, gangliosides and other PL alter lipid composition in the developing brain supporting its structural and functional development. For example, SM supplementation was shown to result in improved neurobehavioral development in low birth-weight infants (60) and a mixture of neuroactive nutrients including SM was recently shown to improve myelination in healthy term infants (61). There are, however, also studies reporting infant plasma or erythrocyte SM or PL levels to remain unaffected by MFGM supplemented IF (62), and in a recent preclinical study in rodents, plasma lipidome changes after dietary MFGM supplementation were not paralleled by significant changes in brain lipidome (63). Other preclinical studies have shown variable effects of dietary MFGM (components) supplementation on brain lipid profile and ganglioside content (64–66), which suggests effects may be dependent on, e.g., concentration and duration of supplementation, brain region studied and methodology used for lipid analysis. Further research on the effects of dietary MFGM supplementation on circulating lipid components and brain lipid composition is warranted.

Another important difference between Concept IF and Standard IF in this study is the structural properties of the lipid droplets, i.e., the large, milk PL-coated lipid droplets in Concept IF versus small lipid droplets without milk PL coating in Standard IF (10). Size and surface area complexity of lipid droplets are important determinants of digestion and absorption kinetics in the gastrointestinal tract (14–18). For example, it was previously demonstrated that consumption of IF with large, PL-coated lipid droplets, similar to the Concept IF used in the current study, results in a different appearance of lipids and postprandial hormones in the circulation (67). It is hypothesized that differences in bioavailability of these components to the developing brain early in life may contribute to infant neurocognitive development (7). This idea is supported by a recent series of preclinical studies in mice demonstrating IF with large lipid droplets coated by PL promote n-3 LCPUFA accumulation in brain tissue and improve later in life cognitive function compared to IF with small lipid droplets and with PL present as added ingredient (21). Interestingly, the mere addition of dairy lipids and/or MFGM components to IF does not lead to structural properties of lipids similar to HM (68), nor does it appear to bring digestion and absorption kinetics closer to that of HM (69). Taken together, the aforementioned findings suggest that IFs containing dairy lipids and/or MFGM (components) as ingredients may help bring neurodevelopment and function of formula fed infants closer to that observed in breastfed infants, and that mimicking of HM with regards to the structural properties of lipid globules may provide additional health benefits for formula fed infants that cannot be reached by composition alone.

Strengths of this study include the long-term follow up and the repeated testing of cognitive function using the same tool at 3, 4, and 5 years of age. Neurocognitive function rapidly develops during early childhood and the NIHTB-CB tests that were applied in the current study allow studying of developmental changes in early childhood (26, 27, 35) that may not be visible when evaluation of neurocognitive function is limited to one timepoint only. Also, the NIHTB-CB was specifically developed for research purposes and is well suited for use in healthy, neurotypical individuals as present in the current study. This is in contrast to some of the more traditionally used test instruments to assess neurocognitive function of young children that have been developed as diagnostic tools to detect neurodevelopmental delay, and that appear to have limited predictive value for neurocognitive function later in life in healthy, neurotypical populations (70). Limitations of the current study are the relatively low sample size, in particular for neurocognitive evaluation at 3 years of age. As at start of the follow-up period no information on the cognitive tests was available in children of 3–5 years of age, analyses were considered of hypothesis generating nature, and no formal power calculations could be performed. Related to the exploratory nature of the investigations, also no multiplicity adjustments were considered. Another aspect is that although the NIHTB-CB is indicated to be suitable for 3–85 years of age, limitations to its utility for measuring executive functions in younger children have been acknowledged (26, 35). Recent evidence suggests that 3 and 4-year-olds may not yet have fully developed the required skills to successfully complete the selected tests (71, 72). In line with this, we observed a relatively high percentage of children not passing the practice trials at 3 and 4 years of age in our study. Some of the differences between the breastfed reference group and formula fed infants in erythrocyte fatty acid composition may be explained by differences in the fatty acid composition between human milk and the study products. For instance, in the current study we observed reduced erythrocyte ALA levels in breastfed infants compared to those of both formula fed groups. Infant circulating ALA levels can be directly associated with dietary ALA supply (73) and ALA levels in human milk are generally lower than the levels provided in IF (41). However, the human milk fatty acid composition was not analyzed in the current study, and thus this hypothesis remains unconfirmed. Also, erythrocyte membrane fatty acid composition might not fully reflect brain fatty acid composition, which could not be analyzed in the current study for obvious reasons. The study was explorative and for this reason results should be interpreted with caution.

In conclusion, this exploratory study suggests, for the first time, that 3–4 months exposure to an IF that closely mimics HM in lipid composition as well as structural properties of lipid droplets during infancy may positively affect cognitive outcomes during childhood. Effects may be mediated by different LCPUFA incorporation in tissue membranes during early life. The structural properties of the lipids in IF, i.e., the large, milk PL-coated lipid droplets are thought to be responsible, at least in part, for the observed effects. IFs mimicking HM with regard to the structural properties of lipid globules may narrow the gap between breastfed and formula-fed infants in neurocognitive development and other health outcomes.

## Data availability statement

The original contributions presented in this study are included in the article/Supplementary material, further inquiries can be directed to the corresponding author.

## Ethics statement

The studies involving humans were approved by the Netherlands Central EC approval: Independent Review Board Nijmegen, Nijmegen, NL Medisch Ethische Toetsings Commissie Erasmus MC, Rotterdam, NL Adviescommissie Mensgebonden Onderzoek Amphia (AMOA), Breda, NL Medisch Ethische Toetsingscommissie (METC) Twente, Enschede, NL METC ISALA, Zwolle, NL Wetenschappelijk Onderzoek Advies Commissie (WOAC), Dordrecht, NL BELGIUM Central EC approval: Commissie Medische Ethiek UZ Brussel, Brussel, BE Ethical Committee ASZ Aalst, Aalts, BE Ethical Committee Clinique et Maternite de Sainte Elisabeth, Namen, BE CHR-Citadelle Ethics Committee, Luik, BE SINGAPORE Central EC approval: SingHealth Centralised Institutional Review Board (CIRB), Singapore. The studies were conducted in accordance with the local legislation and institutional requirements. Written informed consent for participation in this study was provided by the participants’ legal guardians/next of kin.

## Author contributions

SS and NB contributed to the conceptualization, design, and coordination of the study. YV and AH-K collected clinical data. MM-P conducted the statistical analysis. LS wrote the initial draft of the manuscript. All authors contributed to the methodology, investigation, discussion, interpretation of the results, manuscript revision, and read and approved the submitted version of the manuscript.
