# Supplementary material for: Infant formula containing large, milk phospholipid-coated lipid droplets and dairy lipids affects cognitive performance at school age
Source: Front Nutr. 2023 Sep 5;10:1215199. doi: 10.3389/fnut.2023.1215199 (PMC10508340; doi:10.3389/fnut.2023.1215199)
Supplement: Supplementary file 1 [file Table_1.docx]

Supplementary Material

Infant formula containing large, milk phospholipid-coated lipid droplets and dairy lipids affects cognitive performance at school age

Lidewij Schipper^1*^, Nana Bartke^1^, Maya Marintcheva-Petrova^1^, Stefanie Schoen^1^, Yvan Vandenplas^2^, Anita C.S. Hokken-Koelega^3^

^1^ Danone Nutricia Research, Utrecht, The Netherlands

^2^ Universitair Ziekenhuis (UZ) Brussel, Brussel, Belgium

^3^ Erasmus Medisch Centrum -Sophia Kinderziekenhuis, Rotterdam, The Netherlands

*** Correspondence:**Lidewij Schipper
lidewij.schipper@danone.com

# Supplementary Table 1. Fatty acid composition of the intervention products

|  |  | Per 100 ml | Standard IF | Concept IF |
| --- | --- | --- | --- | --- |
| Fatty acids | | g | 3.4 | 3.4 |
| Saturated (SFA) | | g | 1.5 | 1.4 |
|  | C4:0 Butyric | mg | 1.4 | 45 |
|  | C6:0 Caproic | mg | 6.5 | 31 |
|  | C8:0 Caprylic | mg | 64 | 38 |
|  | C10:0 Capric | mg | 49 | 58 |
|  | C12:0 Lauric acid | mg | 374 | 169 |
|  | C14:0 Myristic | mg | 157 | 221 |
|  | C16:0 Palmitic (PA) | mg | 580 | 566 |
|  | of which sn-2 PA | mg | 0 | 202 |
|  | C18:0 Stearic | mg | 104 | 199 |
|  | C20:0 Arachidic | mg | 11 | 10 |
|  | C22:0 Behenic | mg | 8.6 | 9.9 |
|  | C24:0 Lignoceric acid | mg | 2.4 | 2.8 |
| Monounsaturated (MUFA) | | g | 1.3 | 1.2 |
|  | C18:1n-9 Oleic | mg | 1204 | 1111 |
|  | C20:1n-9 Eicosaenoic | mg | 16 | 14 |
|  | C22:1n-9 Erucic | mg | 3.5 | 3.2 |
|  | C24:1n-9 Nervoic | mg | 1.9 | 1.8 |
| Polyunsaturated (PUFA) | | g | 0.6 | 0.6 |
|  | C18:2n-6 Linoleic (LA) | mg | 447 | 447 |
|  | C18:3n-3 Alpha-linolenic (ALA) | mg | 82 | 83 |
|  | C18:3n-6 Gamma linolenic | mg | 0.8 | 0.8 |
|  | C18:4n3 Stearidonic acid | mg | 0 | 2.3 |
|  | C20:3n-6 Dihomo gamma-linolenic (DGLA) | mg | 0.8 | 1.2 |
|  | C20:4n-6 Arachidonic acid (ARA) | mg | 11 | 12 |
|  | C20:5n-3 Eicosapentaenoic (EPA) | mg | 1.4 | 1.8 |
|  | C22:5n-3 Docosapentaenoic (n3DPA) | mg | 0.3 | 1 |
|  | C22:6n-3 Docosahexaenoic acid (DHA) | mg | 6.4 | 6.6 |
