# Supplementary material for: Infant formula containing large, milk phospholipid-coated lipid droplets and dairy lipids affects cognitive performance at school age
Source: Front Nutr. 2023 Sep 5;10:1215199. doi: 10.3389/fnut.2023.1215199 (PMC10508340; doi:10.3389/fnut.2023.1215199)
Supplement: Supplementary file 2 [file Table_2.docx]

Supplementary Material

Infant formula containing large, milk phospholipid-coated lipid droplets and dairy lipids affects cognitive performance at school age

Lidewij Schipper^1*^, Nana Bartke^1^, Maya Marintcheva-Petrova^1^, Stefanie Schoen^1^, Yvan Vandenplas^2^, Anita C.S. Hokken-Koelega^3^

^1^ Danone Nutricia Research, Utrecht, The Netherlands

^2^ Universitair Ziekenhuis (UZ) Brussel, Brussel, Belgium

^3^ Erasmus Medisch Centrum -Sophia Kinderziekenhuis, Rotterdam, The Netherlands

*** Correspondence:**Lidewij Schipper
lidewij.schipper@danone.com

# Supplementary Table 2. Demographics

|  |  | Infants with erythrocyte fatty acid composition data | | | Children with NIH toolbox test results | | |
| --- | --- | --- | --- | --- | --- | --- | --- |
| Characteristics |  | Standard  (n = 25) | Test  (n = 31) | Breastfed  (n = 25) | Standard  (n = 50) | Test  (n = 51) | Breastfed  (n = 50) |
| Child sex | female, % | 72 | 45.2 | 56 | 50 | 51 | 52 |
| Gestational age (weeks) | mean (SD) | 39.06 (1.20) | 39.20 (1.10) | 39.67 (1.36) | 39.10 (1.12) | 39.40 (1.19) | 39.68 (1.29) |
| Cesarean delivery | % | 28 | 41.9 | 28 | 30 | 35.3 | 30 |
| twin | % | 8 | 6.5 | 0 | 8 | 7.8 | 0 |
| Birth weight (g) | mean (SD) | 3231.64 (393.03) | 3252.26 (366.49) | 3401.08 (353.67) | 3249.46 (338.78) | 3341.14 (3429.41) | 3408.12 (342.34) |
| Birth length (cm) | mean (SD) | 49.26 (1.84) | 49.46 (1.57) | 49.87 (1.91) | 49.29 (1.98) | 49.52 (1.58) | 49.95 (1.81) |
| Birth head circumference (cm) | mean (SD) | 34.24 (1.12) | 34.38 (1.21) | 34.32 (1.48) | 34.58 (1.09) | 34.49 (1.24) | 33.99 (1.32) |
| Maternal age (years) | mean (SD) | 30.84 (5.01) | 30 (4.65) | 32.36 (5.50) | 30.3 (4.71) | 31.43 (4.06) | 31.56 (4.82) |
| Maternal education | primary school (N) | 8 | 0 | 0 | 6 | 2 | 0 |
|  | high school/ trade school or equivalent (N) | 52 | 64.5 | 28 | 52 | 58.8 | 38 |
|  | university or higher (N) | 40 | 35.5 | 72 | 42 | 39.2 | 62 |
| Maternal smoking during pregnancy | yes, % | 20 | 19.4 | 0 | 16 | 15.7 | 2 |
